# Genome-wide differential expression of synaptic long noncoding RNAs in autism spectrum disorder

**DOI:** 10.1038/tp.2015.144

**Published:** 2015-10-20

**Authors:** Y Wang, X Zhao, W Ju, M Flory, J Zhong, S Jiang, P Wang, X Dong, X Tao, Q Chen, C Shen, M Zhong, Y Yu, W T Brown, N Zhong

**Affiliations:** 1 Department of Child Health Care, Shanghai Children's Hospital, Shanghai Jiaotong University, Shanghai, China; 2 Chinese Alliance of Translational Medicine for Maternal and Children's Health, Beijing, China; 3 Peking University Center of Medical Genetics, Beijing, China; 4 Department of Human Genetics, New York State Institute for Basic Research in Developmental Disabilities, Staten Island, NY, USA; 5 Student volunteer, Hunter College High School, New York, NY, USA; 6 Department of Obstetrics and Gynecology, Nanfang Hospital, Southern Medical University, Guangzhou, China; 7 March of Dimes Global Network for Maternal and Infant Health, White Plains, NY, USA

## Abstract

A genome-wide differential expression of long noncoding RNAs (lncRNAs) was identified in blood specimens of autism spectrum disorder (ASD). A total of 3929 lncRNAs were found to be differentially expressed in ASD peripheral leukocytes, including 2407 that were upregulated and 1522 that were downregulated. Simultaneously, 2591 messenger RNAs (mRNAs), including 1789 upregulated and 821 downregulated, were also identified in ASD leukocytes. Functional pathway analysis of these lncRNAs revealed neurological pathways of the synaptic vesicle cycling, long-term depression and long-term potentiation to be primarily involved. Thirteen synaptic lncRNAs, including nine upregulated and four downregulated, and 19 synaptic mRNAs, including 12 upregulated and seven downregulated, were identified as being differentially expressed in ASD. Our identification of differential expression of synaptic lncRNAs and mRNAs suggested that synaptic vesicle transportation and cycling are important for the delivery of synaptosomal protein(s) between presynaptic and postsynaptic membranes in ASD. Finding of 19 lncRNAs, which are the antisense, bi-directional and intergenic, of *HOX* genes may lead us to investigate the role of *HOX* genes involved in the development of ASD. Discovery of the lncRNAs of *SHANK2-AS* and *BDNF-AS*, the natural antisense of genes *SHANK2* and *BDNF*, respectively, indicates that in addition to gene mutations, deregulation of lncRNAs on ASD-causing gene loci presents a new approach for exploring possible epigenetic mechanisms underlying ASD. Our study also opened a new avenue for exploring the use of lncRNA(s) as biomarker(s) for the early detection of ASD.

## Introduction

Autism spectrum disorder (ASD) has a reported prevalence of 1 in 68 children in the United States.^1^ ASD is a grouping of lifelong neurodevelopmental disorders, characterized by impairments in reciprocal social interaction and communication, and the presence of stereotypical behaviors, interests or activities. The etiology of ASD is not yet well understood. Although mutations of many genes, including *NLGN3, NLGN4, NRXN1, SHANK2, SHANK3 and PTCHD1*, have been associated with ASD,^2, 3^ metabolic, infectious, inflammatory and other environmental factors have also been implicated in the pathogenesis of ASD.^4, 5, 6, 7, 8, 9^ We previously determined that hypermethylation of the *ENO2* gene is present in 15% of children with ASD,^10^ indicating that epigenetic factor(s) may contribute to the etiology of ASD.^3, 11^ In addition, as transcriptional and posttranscriptional regulators, both microRNAs (miRNAs) and long noncoding RNAs (lncRNAs) have been reported to be involved in ASD as well as in many other neurological disorders.^12, 13, 14, 15, 16, 17, 18, 19, 20, 21^ Overexpression and knockdown studies have shown that lncRNAs have important roles in regulating a variety of processes, including splicing,^22^ transcription,^23^ localization^24^ and the organization of subcellular compartments.^25, 26, 27, 28, 29, 30, 31^ Underscoring the importance of lncRNAs' regulatory roles is their emergence as essential components in the etiology of many disorders, and of complex diseases in particular, for which genetic and environmental interactions have key roles.^31, 32, 33, 34^

LncRNAs are a subset of RNA molecules greater than 200 nt in length that are transcribed but not translated. They may be positioned in genomic sequences as antisense, intronic and large intergenic noncoding RNAs (ncRNAs), as well as at promoter-associated and untranslated regions, which function as *cis* or *trans* regulators. LncRNAs can function as translational and posttranslational regulators of brain development and differentiation, and are associated with various human brain disorders.^16, 17, 18, 19, 20, 21, 35, 36, 37, 38, 39, 40^ LncRNAs have been reported to be involved in many complex diseases, including neurodegenerative and psychiatric diseases, cardiovascular disease, immune dysfunction and auto-immunity, carcinogenesis and reproductive diseases.^41, 42, 43, 44, 45, 46, 47, 48, 49, 50^ Deregulation of lncRNAs is becoming recognized as a major feature of many types of diseases. Importantly, cancer-associated lncRNAs may serve as diagnostic or predictive biomarkers and provide targets for new therapeutic strategies for selective silencing.^51^ Among 168 human diseases that have been found to be associated with lncRNAs, and that are recorded in the *lncrnadisease* (http://cmbi.bjmu.edu.cn/lncrnadisease) database, neurological diseases, cardiovascular diseases and cancers account for 8.3%, 10.7% and 40.5%, respectively.^52^

Altered lncRNA levels have been identified in ASD brains.^21, 53, 54, 55^ In one study of ~33 000 annotated lncRNAs and 30 000 messenger RNA (mRNA) transcripts from the postmortem prefrontal cortex and cerebellar tissues of two ASD and two control subjects, over 200 differentially expressed lncRNAs were detected. These differentially expressed lncRNAs in the ASD subjects were enriched for genomic regions containing genes related to neurodevelopment and neuropsychiatric diseases. Comparison of differences in the expression of mRNAs between the prefrontal cortex and the cerebellum within individual ASD brains showed more transcriptional homogeneity than within control brains. This finding was also true of the lncRNA transcriptome.^55^ Abnormalities in mRNA expression in ASD have also been observed in peripheral blood mononuclear cells, which are safely and easily assayed in infants and offer the potential of a peripheral blood-based, early biomarker panel to detect risk for ASD in infants and toddlers.^56^ We undertook this study to determine whether lncRNAs are differentially expressed in the blood of individuals with ASD, rather than in ASD brains. Our positive findings may open a new approach to investigate potential epigenetic mechanisms underlying ASD and to explore biomarker identifications for possible clinical screening and diagnosis of ASD.

## Materials and methods

### Ethics statement

The Hospital Ethics Committee reviewed and approved the research project. Informed consent was obtained from the parents of the participating children. All the material and data were previously de-identified and coded, and were anonymous to the investigators.

### Subjects

Twenty-five pairs of gender- and age-matched Chinese ASD and control children were recruited for this discovery study at their first-time clinical visit before any clinical laboratory studies, intervention or medication. The children with ASD were clinically diagnosed by means of DSM-IV criteria and did not have epilepsy, any physical disabilities or family history of ASD. The controls were phenotypically and developmentally normal children who were undergoing an annual health checkup. There were 17 pairs of boys and eight pairs of girls, 3–5 years of age, in both the ASD and control groups. Lymphocytes were isolated from 3 to 5 ml of peripheral blood specimens of the Caucasian participants and stored at −70 °C until total RNA was extracted with a Qiagen Mini kit (Qiagen, Valencia, CA, USA). In addition, total RNAs, isolated from 10 lymphoblast cell lines derived from Caucasian children (seven boys and three girls, aged 3 to 8 years) with ASD, were subjected to the validation study. There were no Caucasian control samples used for the validation.

### Microarray hybridization

The Arraystar Human LncRNA Array v2.0 (www.arraystar.com), which detects genome-wide lncRNAs and mRNAs simultaneously, was used for this study. This array covers 33 045 lncRNAs and 30 218 mRNAs that were identified from authoritative data sources, including RefSeq, UCSC Knowngenes and Ensembl. RNA labeling and array hybridizations were performed according to the Agilent One-Color Microarray-Based Gene Expression Analysis protocol (Agilent Technologies, Santa Clara, CA, USA) with minor modifications. Briefly, mRNA was purified from total RNA after the removal of ribosomal RNA with the mRNA-ONLY Eukaryotic mRNA Isolation Kit (Epicentre, Omaha, NE, USA). Each sample was amplified and transcribed into fluorescent complementary RNA (cRNA) along the entire length of the transcripts without a 3′ bias, utilizing the random priming method. The labeled cRNAs were purified using an RNeasy Mini Kit (Qiagen). The concentration and specific activity of the labeled cRNAs (pmol Cy3 per μg cRNA) were measured with NanoDrop ND-1000. One microgram of each labeled cRNA was fragmented by adding 5 μl of 10 × blocking agent and 1 μl of 25 × fragmentation buffer, and then heating the mixture to 60 °C for 30 min. Finally, 25 μl 2 × GE of hybridization buffer was added to dilute the labeled cRNA. Fifty microliters of hybridization solution was dispensed onto the gasket slide and assembled with the lncRNA expression microarray slide. The slides were incubated for 17 h at 65 °C in an Agilent hybridization oven. The hybridized arrays were washed, fixed and scanned by using the Agilent DNA Microarray Scanner (Agilent Technologies). Agilent Feature Extraction software (version 11.0.1.1) was used to analyze the acquired array images. Quantile normalization and subsequent data processing were performed using the GeneSpring GX v12.1 software package (Agilent Technologies). After normalization of the raw data, lncRNAs and mRNAs that had flags (‘All Targets Value') were chosen for further data analysis. Differentially expressed lncRNAs and mRNAs between the two groups with statistical significance were identified through volcano plot filtering. Hierarchical clustering was performed using the Agilent GeneSpring GX software (Version 12.1). Both ‘GO analysis' and ‘Pathway analysis' were performed with the DAVID program (http://david.abcc.ncifcrf.gov), in which analysis of gene ontology (GO) and KEGG PATHWAY was conducted. The results were also analyzed using the genetic and molecular interaction software GeneMANIA,^57, 58^ an algorithm to determine the relationship between these mRNAs. The bio-functions and canonical pathways associated with our data were generated by using the core-analysis option in Ingenuity Pathway Analysis (Ingenuity Systems; http://www.ingenuity.com).

### Quantitative real-time PCR analysis

The total RNA extracted from leukocytes or from lymphoblasts was used to synthesize cDNA. The expression levels of lncRNAs and of lncRNA-targeted mRNAs were determined by quantitative real-time PCR. Quantitative PCR reactions (the primer sequences used in quantitative PCR are listed in Supplementary Table S1) were performed by the ABI7900 system (Life Technologies, Grand Island, NY, USA) and SYBR green dye SuperArray PCR master mix (SABiosciences, Frederick, MD, USA). mRNA of glyceraldehyde-3-phosphate dehydrogenase (GAPDH) was used as an internal control for quantitative analysis of lncRNA or mRNA. The lncRNA or mRNA values were normalized to GAPDH levels. For each lncRNA or mRNA, triple reactions were analyzed simultaneously, and the result was reported as the relative expression calculated relative to this control. All the data were given in terms of relative expression of the mean±S.E. (*n*=10). The data were subjected to one-way analysis of variance followed by an unpaired, two-tailed *t*-test. Differences were considered significant at *P*<0.05.

## Results

### Differential expression profiles of lncRNA and mRNAs

A total of 3929 lncRNAs were identified as differentially expressed in Chinese ASD peripheral blood cells, including 2407 that were upregulated and 1522 that were downregulated. Among these, intergenic lncRNAs were the most common (accounting for 43%), followed by natural antisense (19%), intronic antisense (12%), exon sense-overlapping (9%), bi-directional (5%) and intron sense-overlapping (4%). Five percent of identified lncRNAs belong to uncharacterized groups (Supplementary Table S2). Simultaneously, 2610 mRNAs, including 1789 upregulated and 821 downregulated, that were differentially expressed in ASD blood cells genome-wide were also identified. The entire data set has been deposited in a public domain (DataDryad.org, DOI: doi:10.5061/dryad.d8f84).

### Functional pathways derived from lncRNAs–mRNAs

**Figure 1:**
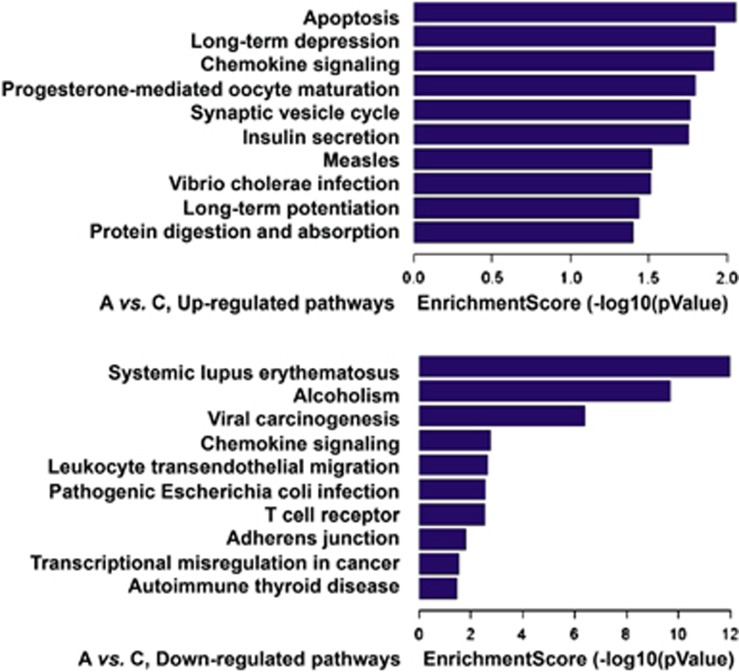
Metabolic pathways characterized from the lncRNAs differentially expressed in ASD: The top-10 score of up- and downregulated pathways were characterized with KEGG functional analysis. Three *P*-values, the EASE-score, Fisher *P-*value and Hypergeometric *P*-value were integrated for the analysis. The bar plot shows the top Enrichment Score [−log10(*P-*value)] value of the significant enrichment pathway. The higher Enrichment Score indicates that more lncRNA molecules are involved in this pathway. ASD, autism spectrum disorder; lncRNA, long noncoding RNA.

The gene loci where the lncRNAs are localized were subjected to pathway and gene ontology analysis. A total of 13 pathways derived from upregulated lncRNAs and 14 from downregulated lncRNAs were identified as being significant in ASD group (*P*<0.05). The 10 pathways with the highest enrichment score (Figure 1) showed that downregulated lncRNA loci were mainly involved in infection and inflammatory pathways. However, three pathways that are related to neurological regulation—the long-term depression, the synaptic vesicle cycling and the long-term potentiation pathways—were characterized from the upregulated lncRNA loci. The plot of enrichment score shows the relevant level of probability of the involvement of differentially expressed lncRNAs in the pathway. The range-axis in the upregulated pathways is lower than in the downregulated, suggesting that the pathogenic impact of lncRNAs in downregulated pathways is heavier than in upregulated pathways and the downregulated pathways are more likely involved in ASD than in upregulated pathways.

### Differential expression of synaptic lncRNAs and mRNAs

**Figure 2:**
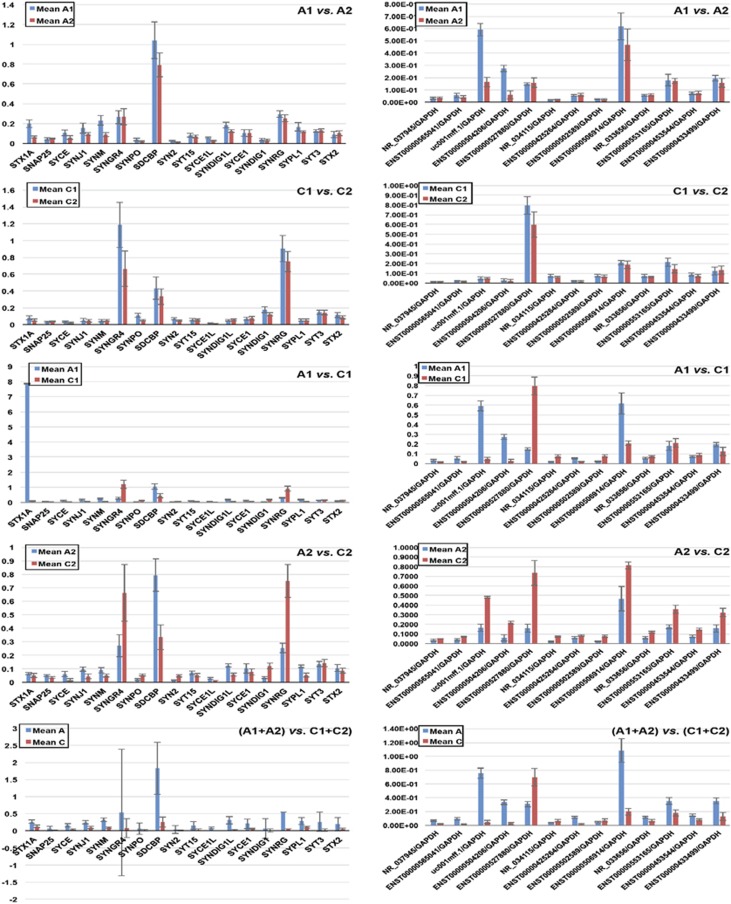
Validation of lncRNAs: qRT-PCR was applied to validate differentially expressed lncRNAs (right panels) and mRNAs (left panels) between the ASD and the control groups. Other than the gene symbol, each lncRNA was labeled with its name that can be matched to the symbol in Table 3. The height of each bar, measured by mean±s.d., represents the relative expression level. ASD, autism spectrum disorder; lncRNA, long noncoding RNA; qRT-PCR, quantitative real-time PCR.

Thirteen synaptic lncRNAs (Table 1A), including nine upregulated and four downregulated, and 19 synaptic mRNAs (Table 1B), including 12 upregulated and seven downregulated, were identified as being differentially expressed in children with ASD. Among the upregulated lncRNAs, six were intronic antisense, two exon sense-overlapping and one natural antisense. Among the downregulated lncRNAs, three were natural antisense and one intronic antisense. To validate the differential expression of the synaptic lncRNAs and mRNAs identified from the microchip-based discovery study, the lncRNAs and mRNAs were subjected to quantitative analysis by quantitative PCR. Before the differential expression between the ASD and the control groups was analyzed, inter-group comparisons were made between the lymphocytes in a Chinese population and the lymphoblasts in a Caucasian population (A1 vs A2, C1 vs C2). As shown in Table 2A, the expression of lncRNAs NR_037945 (STX16), ENST00000565041 (SYNGR3), ENST00000425264 (SLC18A2), ENST00000502589 (SV2C) and ENST00000453544 (SNAP25) showed no significant difference between the Chinese and the Caucasian populations in both the ASD and the control groups; expression of ENST00000527880 (SYP), NR_034115 (STXBP5), NR_033656 (STX8) and ENST00000553165 (SYT1) showed no significant difference between the Chinese ASD groups; and expression of uc001mff.1 (SYT9), ENST00000504206 (SYT9), ENST00000506914 (SYT15) and ENST00000433499 (STXBP5) showed no significant difference between the two control groups. The differential expression of all lncRNAs, except ENST00000453544 (locus SNAP25) in the Caucasian ASD subjects and ENST00000553165 (SYT1) in both the Chinese and Caucasian ASD subjects, was statistically significant between the ASD and control groups. However, there was no statistical significance (*P*>0.05) observed between the males and females, nor between different age groups, in ASD or controls. No significant difference in the expression of three mRNAs—NGR4, SYNDIG1 and STX2—was evident between the Chinese and Caucasian ASD subjects. Expression of the mRNAs SYNJ1, SDCBP, SYPL1, SYNM and SYNDIG1L was not significantly different between the Chinese and Caucasian control groups. Expressions of all mRNAs, except SYCE1 in the Caucasian population and STX2 and SYT3 in both populations, were determined to be significantly different between the ASD and control groups in both the populations (Table 2B). To integrate the genome-wide-expressed lncRNAs with the synaptic mRNAs, we were able to draw the networks between the lncRNAs and mRNAs (Figure 2). This helps associate differential gene expression with gene ontology, biological pathway and the regulatory functions of the lncRNAs.

### Association of lncRNAs with autistic genes

Genetic and genomic studies have revealed that a substantial proportion of ASD risk resides in high-impact rare variation, ranging from chromosome abnormalities, single-nucleotide variation, copy-number variation to gene mutations.^59^ To match our lncRNA results to these gene loci, 19 lncRNAs were found to be associated with ASD genes (Table 3A). Among these lncRNAs, seven were natural antisense (AS); six, intronic antisense; three, bi-directional; two, intron sense-overlapping; and one, intergenic. Twelve of the 19 lncRNAs associated with ASD genes were homeobox or homeobox-related genes, including nine that were upregulated and three that were downregulated, followed by four brain-derived neurotrophic factor (BDNF) isoforms. Interestingly, differential expression of mRNAs for *HOXA* and *HOXB* was also identified in our microarray-based discovery study of the ASD patients (Table 3B). However, no mRNAs, which are the targets of *BDNF-AS* and of the intronic AS of *SHANK2*—a membrane of the SHANK gene family and its gene mutations found in ASD patients (http://autism.mindspec.org/GeneDetail/SHANK2)—were identified.

## Discussion

### Differentially expressed lncRNAs represent a new potential biomarker category for the early detection of ASD

Previous studies have reported that lncRNAs were aberrantly expressed in brain tissues and associated with ASD.^56^ However, brain tissue cannot be used as clinical material for early screening or for diagnostic purposes. In addition, there is no specific biomarker at present that can be applied in clinical practice, owing to the genetic heterogeneity of ASD,^10^ although efforts have been undertaken to characterize blood mRNA profiles.^60, 61, 62, 63^ Use of a panel of lncRNAs that are specifically associated with ASD phenotypes and are differentially expressed in ASD peripheral blood would be valuable and practical. In this study, we presented metabolic pathways (Figure 1) that define peripheral blood lncRNAs that are differentially expressed in ASD. Among these, synaptic vesicle cycling, long-term depression and long-term potentiation are neurologically related pathways. LncRNAs that are differentially expressed in ASD and have been identified in these pathways include IGF1, mGluR1, CRFR1, IGF1R, NMDAR and VDCC, which are localized to cell membranes; and Ras, G protein, PLC, IP3R, PKG, ERK1, ERK2 and PP2A, which are in the cytoplasm and involved in signal transduction for long-term depression and long-term potentiation. The differentially expressed lncRNAs involved in the synaptic vesicle cycling pathway are Rab3A, Munc13, Syntaxin, SNAP25, Clathrin, V-ATPase and trans-SNARE complex. Expression of all these genes is found with various technology platforms (www.genecards.org). It is not clear whether the lncRNAs and mRNAs identified in the peripheral lymphocytes are identical to those expressed in neuronal cells or whether they reflect ASD brain functions.

Although lncRNAs have been determined to associate with transcriptional regulation in neuronal development and diseases,^17^ applying gene differential expression profile analysis of peripheral bloods for brain disorders presents a challenge to convince that the differential expression profile in the peripheral bloods may be relevant to that in brain tissues. In fact, there is no way to obtain human brain tissue for routine screening or diagnostic analysis in clinical practice. The eQTL gene transcripts identified from brains was demonstrated as being stably expressed in peripheral bloods.^64, 65^ Our earlier study also determined that the brain gene *ENO2* showed differentially expressed methylation in peripheral bloods.^10^ Therefore, to analyze differential expression profile in blood may open a new approach to explore applying differential expression profile in blood as a biomarker for brain diseases.

The differential expression we found demonstrates the potential for lncRNAs to be applied as clinical biomarkers. Replication of our study with larger samples and various ethnic backgrounds will be needed. Indeed, we noted differences in the lncRNAs when comparing the Chinese and Caucasian populations (Table 2A). These differences suggest that there exist, at certain gene loci, inter-population and inter-condition differences in gene expression. A similar finding of the influence of different ethnic backgrounds was also observed in the mRNAs (Table 2B).

### Synaptic lncRNAs may regulate synaptic vesicle transportation

Among the 13 synaptic lncRNAs, three were lncRNAs, which resided at the genes for synaptic vesicle proteins (Table 1A). The gene *SLC18A2*, a member of the vesicular monoamine transporter family, encodes a vesicular monoamine transporter of cytosolic monoamines into synaptic vesicles, using the proton gradient maintained across the synaptic vesicular membrane. Its proper function is essential for the proper activity of monoaminergic systems, which have been implicated in several human neuropsychiatric disorders, including brain dopamine–serotonin vesicular transport disease and cocaine dependence.^66^ The gene *SV2C* encodes synaptic vesicle glycoprotein 2C, which has a role in the control of regulated secretion in neural and endocrine cells, selectively enhances low-frequency neurotransmission, and positively regulates vesicle fusion by maintaining the readily releasable pool of secretory vesicles.^67^ *SYP* is a gene that encodes the synaptic protein synaptophysin, an integral membrane protein of small synaptic vesicles in the brain and endocrine cells, which is a transporter and a calcium ion–binding protein. This protein may also bind cholesterol and is thought to direct targeting of vesicle-associated membrane protein 2 (synaptobrevin) to intracellular compartments.^68^ Mutations in this gene are associated with X-linked mental retardation (www.researchgate.net/publication/12901506_XLMR_database).^69^ In addition to these three synaptic vesicle proteins, the genes *STX8* and *STX16* are also involved in synaptic vesicle metabolism. The *STX8* gene is involved in protein trafficking from early to late endosomes via vesicle fusion and exocytosis. It encodes a vesicle trafficking protein that functions in the early secretory pathway, possibly by mediating retrograde transport from *cis*-Golgi membranes to the endosome reticulum.^70, 71^ The *STX16* gene is a member of the t-SNARE (target-SNAP receptor) family. Proteins in this family are found on cell membranes and serve as the targets for V-SNARES (vesicle-SNAP receptors), permitting specific synaptic vesicle docking and fusion. A disease associated with *STX8* includes visual epilepsy, and diseases associated with *STX16* are pseudohypoparathyroidism type 1b and pseudohypoparathyroidism.^72, 73^

### *HOX* genes are likely to be deregulated in ASD

Several studies have demonstrated that lncRNAs can function in the regulation of *in vivo* transcription. An lcnRNA dubbed linc-HOXA1 RNA has been found to repress *Hoxa1* expression. Knockdown of linc-HOXA1 increases transcription of the *Hoxa1* gene that is located some 50 kb adjacent to the linc-HOXA1.^74^ *HOXA* cluster antisense RNA 2 (HOXA-AS2) is an lncRNA located between the *HOXA3* and *HOXA4* genes in the *HOXA* cluster. HOXA-AS2 is an apoptosis repressor in all *trans* retinoic acid-treated NB4 promyelocytic leukemia cells.^75^ Its transcript is expressed in NB4 promyelocytic leukemia cells and human peripheral blood neutrophils, and expression is increased in NB4 cells treated with all *trans* retinoic acid. The all *trans* retinoic acid induction of HOXA-AS2 suppresses all *trans* retinoic acid-induced apoptosis.^75^ The *HOTAIR* (Hox transcript antisense RNA) gene contains 6232 bp and encodes a 2.2- kb lncRNA. Its source DNA is located within a *HOXC* gene cluster. Recently, differential expression of *HOTAIR* has been determined to be associated with cancer metastasis and possibly to represent an independent prognostic factor.^76^ The 5′ end of *HOTAIR* interacts with the polycomb-group protein Polycomb repressive complex 2 and as a result regulates chromatin state. It is required for gene-silencing of the *HOXD* locus by Polycomb repressive complex 2 and the 3′ end of *HOTAIR* interacts with the histone demethylase LSD1.^77^ In our study, we identified several lncRNAs of the *HOX* genes (*HOXA13*, *HOXB5*, *HOXB6* and *HOXD1*) and *HOX*-related genes (*DLX6*, *HMBOX1* and *BARX1*) from leukocytes derived from ASD patients (Table 3A). These findings led us to hypothesize that the differentially expressed lncRNAs of *HOX* genes and *HOX*-related genes, referred to as lncHOXs, could represent a new set of biomarkers for ASD.

The variety of identified lncRNAs suggests that lncRNAs' regulatory functions involved in ASD may have various epigenetic mechanisms.

LncRNAs have been recognized as transcriptional and posttranscriptional regulators.^28, 29, 30, 31, 32, 33^ They may function to activate gene transcription by binding a transcriptional factor to the promoter region to signal or guide transcription. They may prevent miRNA from binding to the target gene, may suppress gene transcription by decoying the transcription factor away from the promoter, or may be a chromatin modifier by bringing a chromatin enzyme onto chromatin to form a complex and thereby modify histones.^28, 29, 30, 31, 32, 33^ Usually, if the lncRNA is the antisense of a gene, the lncRNA likely functions as the *cis*-suppressor to inhibit gene transcription. This could be the case for both the natural and intronic antisense lncRNAs that we identified (Tables 1A and 3A). To further understand their molecular mechanisms, transgenic models created by introducing extragenic lncRNA to generate a knockout, knockin or knockdown model at the cellular and/or animal level could be investigated. Such a transgenic model could provide phenotype(s) to mimic ASD. So far, there is little evidence that details the molecular and pathogenic mechanisms of the bi-directional and intergenic lncRNAs involved in ASD and other neurological diseases. In the present study, we identified three bi-directional and one intergenic lncRNA, and observed that all are located within *HOX* loci (Table 3A). A better understanding of the molecular mechanisms will clarify how these lncRNAs are involved in regulating gene expression in ASD and other neurological conditions.

In conclusion, we have profiled here the differential expression of lncRNAs and mRNAs in ASD peripheral leukocytes and have identified important clusters that may be associated with this disorder. Our findings suggest the importance of synaptic lncRNAs, which are likely involved in synaptic vesicle transportation and cycling and thus would be important for the delivery of synaptosomal protein(s) between presynaptic and postsynaptic membranes. LncRNAs that are the antisense of the *HOX* genes may be related to ASD. This finding may open a new approach to investigate the pathogenic mechanisms of the *HOX* genes in the development of ASD. Identification of the lncRNAs of *SHANK2-AS* and *BDNF-A*S indicate that in addition to gene mutation, de-regulation of lncRNAs on ASD-causing gene loci may represent a new category of, and allow exploration of, the epigenetic mechanisms involved in ASD. Further investigation with larger sample sizes may validate the use of lncRNAs as biomarkers for early detection of ASD.

## Figures and Tables

**Table 1A:** Synaptic lncRNAs differentially expressed in ASD (discovery)

*LncRNA name*	P-*value*	*Fold change*	*Regulation*	*LncRNA symbol*	*Chromosome*	*LncRNA strand*	*Relationship*	*Associated gene name*	*Associated gene strand*	*Associated protein*
ENST00000506914	6.22808E−10	2.986859	Up	RP11-38L15.3	Chr10	+	Intronic antisense	SYT15	−	Synaptotagmin-15 isoform a
NR_037945	4.65294E−09	2.772749	Up	STX16-NPEPL1	Chr20	+	Exon sense-overlapping	STX16	+	Syntaxin-16 isoform
ENST00000565041	3.29675E−09	2.453277	Up	AC005606.14	Chr16	−	Intronic antisense	SYNGR3	+	Synaptogyrin-3
NR_033656	3.16257E−08	2.318933	Up	STX8	Chr17	−	Exon sense-overlapping	STX8	−	Syntaxin-8
ENST00000553165	1.09728E−09	2.007777	Up	RP1-78O14.1	Chr12	−	Intronic antisense	SYT1	+	Synaptotagmin-1
ENST00000453544	2.61069E−09	2.291905	Up	RP5-839B4.7	Chr20	−	Intronic antisense	SNAP25	+	Synaptosomal-associated protein 25 isoform
ENST00000425264	2.99156E−08	2.407836	Up	RP11-501J20.5	Chr10	−	Natural antisense	SLC18A2	+	Synaptic vesicular amine transporter
uc001mff.1	1.14979E−11	3.098403	Up	AK128569	Chr11	−	Intronic antisense	SYT9	+	Synaptotagmin-9
ENST00000504206	9.3421E−13	2.766950	Up	CTD-2516F10.2	Chr11	−	Intronic antisense	SYT9	+	Synaptotagmin-9
ENST00000502589	8.16875E−10	2.804515	Down	RP11-466P24.2	Chr5	−	Intronic antisense	SV2C	+	Synaptic vesicle glycoprotein 2C
ENST00000527880	5.19056E−22	3.546924	Down	SYP-AS1	ChrX	+	Natural antisense	SYP	+	Synaptic vesicle glycoprotein 2C
ENST00000433499	1.12117E−10	2.567277	Down	STXBP5-AS1	Chr6	−	Natural antisense	STXBP5	−	Synaptophysin
NR_034115	3.93428E−12	3.163574	Down	STXBP5-AS1	Chr6	−	Natural antisense	STXBP5	+	Syntaxin-binding protein 5 isoform

Abbreviations: ASD, autism spectrum disorder; lncRNA, long noncoding RNA.

**Table 1B:** Synaptic mRNAs differentially expressed in ASD (discovery)

*Gene symbol*	P-*value*	*Fold change*	*Regulation*	*LncRNA name*	*Chromosome*	*Gene strand*	*Protein*
SYNDIG1L	2.07E−10	2.4465995	Up	ENST00000331628	chr14	−	Synapse differentiation-inducing 1-like
SYNJ1	1.05E−09	2.0476525	Up	NM_203446	chr21	−	Synaptojanin-1 isoform b
SYCE1	2.58E−09	2.0756986	Up	ENST00000303903	chr10	−	Synaptonemal complex central element protein 1
SYCE1L	2.63E−07	2.1141472	Up	NM_001129979	chr16	+	Synaptonemal complex central element protein 1-like
SYCE2	1.76E−10	2.7053528	Up	NM_001105578	chr19	−	Synaptonemal complex central element protein 2
SYPL1	9.68E−09	2.6788418	Up	NM_006754	chr7	−	Synaptophysin-like protein 1 isoform a
SNAP25	7.44E−11	2.2339847	Up	NM_130811	chr20	+	Synaptosomal-associated protein 25 isoform
SYT15	8.02E−07	2.0689712	Up	ENST00000374325	chr10	−	Synaptotagmin XV
SYT3	7.75E−09	2.019983	Up	NM_001160329	chr19	−	Synaptotagmin-3
SDCBP	4.00E−09	3.5665565	Up	ENST00000260130	chr8	+	Syndecan binding protein (syntenin)
SYNM	7.08E−11	2.8931897	Up	NM_015286	chr15	+	Synemin isoform B
STX1A	6.43E−10	2.8111503	Up	NM_004603	chr7	−	Syntaxin-1A isoform 1
SYNDIG1	1.17E−11	2.1523478	Down	NM_024893	chr20	+	Synapse differentiation-inducing gene protein 1
SYN2	8.58E−09	2.0109727	Down	NM_133625	chr3	+	Synapsin-2 isoform IIa
SYNGR4	4.79E−06	2.0704043	Down	NM_012451	chr19	+	Synaptogyrin-4
SYNJ1	6.97E−12	2.1072302	Down	NM_003895	chr21	−	Synaptojanin-1 isoform a
SYNPO	1.59E−13	2.8214178	Down	NM_001109974	chr5	+	Synaptopodin isoform B
SYNRG	3.89E−17	2.5995717	Down	NM_198882	chr17	−	Synergin gamma isoform 3
STX2	6.70E−10	2.1167502	Down	NM_194356	chr12	−	Syntaxin-2 isoform 2

Abbreviations: ASD, autism spectrum disorder; lncRNA, long noncoding RNA; mRNA, messenger RNA.

**Table 2A:** Synaptic lncRNAs differentially expressed in ASD (validation)

		*NR_037945*	*ENST00000565041*	*ENST00000425264*	*ENST00000502589*	*ENST00000453544*	*ENST00000527880*	*NR_034115*	*NR_033656*	*ENST00000553165*	*uc001mff.1*	*ENST00000504206*	*ENST00000506914*	*ENST00000433499*
A1 vs A2	*P*	0.4559	0.0688	0.1267	0.6145	0.6630	0.3911	0.3517	0.3925	0.7064	0.0000	0.0000	0.0110	0.0128
	*t*	0.762	1.935	1.601	0.513	0.443	0.896	0.966	0.876	0.385	20.982	16.173	2.829	2.764
C1 vs C2	*P*	0.2169	0.0862	0.5017	0.2529	0.272	0.0009	0.013	0.0369	0.0021	0.9356	0.3177	0.1648	0.6184
	*t*	1.28	1.815	0.686	1.181	2.405	3.949	2.756	2.265	3.595	0.082	1.028	1.448	0.507
A1 vs C1	*P*	0.0001	0.0002	0.0000	0.0000	0.0193	0.0000	0.0000	0.0056	0.1089	0.0000	0.0000	0.0000	0.0001
	*t*	5.792	5.646	14.119	11.704	2.569	22.538	12.138	3.197	1.687	31.772	26.686	11.679	4.858
A2 vs C2	*P*	0.0000	0.0000	0.0000	0.0000	0.1370	0.0000	0.0000	0.0297	0.7743	0.0000	0.0000	0.0000	0.0003
	*t*	9.768	9.6730	16.1620	13.3310	1.5660	25.8450	17.5950	2.3720	0.2910	26.2790	22.3500	12.1380	4.4780

Abbreviations: ASD, autism spectrum disorder; A1, Chinese ASD; A2, Caucasian ASD; C1, Chinese control; C2, Caucasian control; lncRNA, long noncoding RNA.

**Table 2B:** Synaptic mRNAs differentially expressed in ASD (validation)

		*SYCE1*	*SYNGR4*	*SYNDIG1*	*SNAP25*	*STX2*	*SYT3*	*SYT15*	*SYNRG*	*SYNJ1*	*SDCBP*	*SYPL1*	*STX1A*	*SYCE2*	*SYNM*	*SYNPO*	*SYN2*	*SYCE1L*	*SYNDIG1L*
A1 vs A2	*P*	0.9354	0.8693	0.4318	0.3603	0.2745	0.2609	0.0707	0.0118	0.0041	0.0023	0.0019	0.0000	0.0004	0.0000	0.0009	0.0000	0.0000	0.0000
	*t*	0.0820	0.1670	0.8040	0.9510	1.1270	1.1610	1.9210	2.8000	3.6080	3.5410	3.6370	10.0110	4.3530	8.7390	3.9480	5.6170	7.9180	5.9400
C1 vs C2	*P*	0.4557	0.0001	0.0008	0.0813	0.0320	0.7826	0.4860	0.0239	0.3098	0.0748	0.9628	0.0035	0.0007	0.5587	0.0000	0.0006	0.0057	0.5068
	*t*	0.7620	4.8350	4.0040	1.8470	2.3250	0.2800	0.7110	2.4660	1.0450	1.8910	0.0470	3.3600	4.0750	0.5510	7.9550	4.1700	3.1400	0.6770
A1 vs C1	*P*	0.0055	0.0000	0.0000	0.0146	0.1238	0.0511	0.0027	0.0000	0.0001	0.0000	0.0000	0.0000	0.0000	0.0000	0.0000	0.0000	0.0000	0.0000
	*t*	3.1560	10.4850	11.4310	2.7870	1.6140	2.0900	3.4830	11.9790	6.1330	8.4260	8.0720	7.7860	7.2810	11.8300	8.6720	9.6360	12.7730	13.4130
A2 vs C2	*P*	0.4620	0.0001	0.0000	0.0001	0.0816	0.5086	0.0076	0.000	0.0000	0.0000	0.0000	0.0155	0.0000	0.0000	0.0000	0.0000	0.0000	0.0000
	*t*	2.1410	5.4670	13.0950	4.9060	1.8440	0.6740	3.0030	12.478	6.9610	9.5700	11.8830	2.6730	6.4040	5.5550	10.0800	10.4070	6.8990	11.9880
A vs C	*P*	0.0090	0.0000	0.0000	0.0000	0.8327	0.0910	0.0015	0.000	0.0000	0.0000	0.0000	0.0000	0.0000	0.0000	0.0000	0.0000	0.0000	0.0000
	*t*	3.1670	14.3460	15.3330	6.6640	0.2140	1.8380	3.7420	17.277	7.6950	11.4130	12.1930	7.1870	8.8100	13.0440	11.0910	15.1610	19.9780	19.7310

Abbreviations: ASD, autism spectrum disorder; A1, Chinese ASD; A2, Caucasian ASD; C1, Chinese control; C2, Caucasian control; mRNA, messenger RNA.

**Table 3A:** LncRNAs associated with autistic genes

*LncRNA name*	P-*value*	*Fold change*	*LncRNA symbol*	*LncRNA strand*	*Relationship*	*Associated gene accession*	*Associated gene name*	*Associated gene strand*	*Associated protein*
*Upregulated*									
ENST00000454594	9.88E−08	2.25	HOTTIP	+	Intronic antisense	NM_000522	HOXA13	−	Homeobox protein Hox-A13
ENST00000476204	2.06E−11	3.41	HOXB-AS3	+	Intronic antisense	NM_002147	HOXB5	−	Homeobox protein Hox-B5
ENST00000477144	6.89E−11	2.09	HOXB-AS3	+	Natural antisense	NM_018952	HOXB6	−	Homeobox protein Hox-B6
ENST00000476204	2.06E−11	3.41	HOXB-AS3	+	Intronic antisense	NM_018952	HOXB6	−	Homeobox protein Hox-B6
ENST00000462131	4.89E−10	2.45	HOXB-AS4	+	Intergenic				
ENST00000452365	2.85E−10	2.79	HOXD-AS1	−	Bidirectional	NM_024501	HOXD1	+	Homeobox protein Hox-D1
ENST00000430404	4.212E−04	2.97	DLX6-AS1	−	Intronic antisense	NM_005222	DLX6	+	Homeobox protein DLX6
ENST00000558292	1.73E−13	3.71	AC108449.2	+	Intron sense-overlapping	NM_001135726	HMBOX1	+	Homeobox-containing protein 1
ENST00000558292	1.73E−13	3.71	AC108449.2	+	Intron sense-overlapping	NM_024567	HMBOX1	+	Homeobox-containing protein 1
ENST00000454594	8.43E−09	2.27	RP11-231K24.2	+	Bidirectional	NM_021570	BARX1	−	Homeobox protein BarH-like 1
NR_033314	9.454E−08	3.30	BDNF-AS	+	Natural antisense	NM_170735	BDNF	−	Brain-derived neurotrophic factor isoform a preproprotein
uc009yim.3	7.610E−09	2.71	BDNF-AS1	+	Natural antisense	NM_001143805	BDNF	−	Brain-derived neurotrophic factor isoform a preproprotein
ENST00000307548	6.98E−09	2.62	SHANK2-AS3	+	Intronic antisense	NM_012309	SHANK2	−	SH3 and multiple ankyrin repeat domains protein 2 isoform 1

*Downregulated*									
ENST00000521231	6.06E−10	3.73	HOXA-AS3	+	Natural antisense	NM_024014	HOXA6	−	Homeobox protein Hox-A6
ENST00000526796	3.83E−08	2.09	RP11-687M24.8	−	Intronic antisense	NM_022062	PKNOX2	+	Homeobox protein PKNOX2
ENST00000526796	1.33E−06	2.53	RP11-687M24.5	−	Bidirectional	NM_022062	PKNOX2	+	Homeobox protein PKNOX2
NR_033315	1.57E−12	2.11	BDNF-AS	+	Natural antisense	NM_001143810	BDNF	−	Brain-derived neurotrophic factor isoform e
uc009yix.3	1.77E−17	2.60	BDNF-AS1	+	Natural antisense	NM_001143805	BDNF	−	Brain-derived neurotrophic factor isoform a preproprotein
ENST00000481143	2.82E−07	2.15	DMD-AS1	+	Natural antisense	NM_004014	DMD	−	Dystrophin Dp116 isoform

Abbreviations: ASD, autism spectrum disorder; BDNF, brain-derived neurotrophic factor; lncRNA, long noncoding RNA.

**Table 3B:** Differential expression of mRNAs encoded by autistic genes

*Gene symbol*	P-*value*	*Fold change*	*Regulation*	*Gene accession*	*ccdsID*	*Chromosome*	*Gene strand*	*Protein*
HOXA1	6.8332E−14	2.2217333	Down	NM_153620	CCDS5402.2	Chr7	−	Homeobox protein Hox-A1 isoform b
HOXA4	8.80085E−16	2.0322213	Down	NM_002141	CCDS5405.1	Chr7	−	Homeobox protein Hox-A4
HOXB2	1.45676E−15	2.055423	Down	NM_002145	CCDS11527.1	Chr17	−	Homeobox protein Hox-B2
HOXB4	2.47971E−07	2.034943	Down	NM_024015	CCDS11529.1	Chr17	−	Homeobox protein Hox-B4
HOXB8	1.37338E−09	2.101062	Down	ENST00000239144	CCDS11533.1	Chr17	−	Homeobox B8

Abbreviation: mRNA, messenger RNA.
