# Supplementary material for: Genome-wide differential expression of synaptic long noncoding RNAs in autism spectrum disorder
Source: Transl Psychiatry. 2015 Oct 20;5(10):e660–. doi: 10.1038/tp.2015.144 (PMC4930123; doi:10.1038/tp.2015.144)
Supplement: Supplementary Tables [file tp2015144x1.pdf]

Table S1. Primer sequences of qPCR

lncRNA	Primers	Tm (°C)	Amplicon (bp)
GAPDH	F:5'GGGAAACTGTGGCGTGAT3'<br>R:5'GAGTGGGTGTCGCTGTTGA3'	60	299
ENST00000506914	F:5' GAAAGAAGGACGAGGGATTGG3'<br>R:5' GGAGTGTGACTGCGGAGGC3'	60	273
NR_037945	F:5' GGGCACGGAAACGAATACAG3'<br>R:5' CCGAGTTCTCAGCCAAGCAG3'	60	198
ENST00000565041	F:5' GGACCCTCAGAAAGACCCGA3'<br>R:5' GGCCATTGCCTTCCCACTA3'	60	136
NR_033656	F:5' CTGCTTGTGGCTATCGTGGT3'<br>R:5' TTTTTCAGAGCTTTGGGGGA3'	60	156
ENST00000553165	F:5' CTGCTCTGCTGAGACTCCACA3'<br>R:5' TTTTCCCCACTCCATCCTTC3'	60	109
ENST00000453544	F:5' TTTGTGTGTACCGAGTTTGGGA3'<br>R:5' GGATTCATGAGGCATTTTTGC3'	60	255
ENST00000425264	F:5' CGCTCTCTCCACCTCCCTACT3'<br>R:5' GCCATCTGCTTCCTCCTCCTA3'	60	194
uc001mff.1	F:5' GACATGTTTGGGTGCCAGTTAT3'<br>R:5' GGACAGGAGGTTTCCAGTGATT3'	60	162
ENST00000504206	F:5' GAGATGGTAGGCACCGTGAA3'<br>R:5' TGAGCAGAAGGCTGGAGAGA3'	60	155
ENST00000502589	F:5' TTGCTTTTCCGTCTTCCCG3'<br>R:5' CAGCTTCCCAGTGCTGTGGT3'	60	167
ENST00000527880	F:5' CTGTGAATCCCTTGCTTGCTC3'<br>R:5' AATACTGTGGCTCCCTGTTGG3'	60	295
ENST00000433499	F:5' AACTGACCCCCAGCCACC3'<br>R:5' CTTCTCACCCTCTCCCCGA3'	60	141
NR_034115	F:5' CTTCACCACACAACTCACACTCTC3'<br>R:5' ATTCCTTTCCACCCAACCTAAC3'	60	212
**lncRNA**	**Primers**	**Tm (°C)**	**Amplicon (bp)**
STX1A	F:5' CAGCTTCCCTTCCCACATTC3'<br>R:5' CAAGACAACAGGAGCCACCA3'	60	192
SNAP25	F:5' TTGGTGGCTCTAACTCCTTGA3'<br>R:5' AAGGACCGTGGCAGTAACTCT3'	60	187
SYCE2	F:5' AGCCTGAGCAGAGCCTAAGAC3'<br>R:5' CAGCCACAGAAGAAACGAACA3'	60	111
SYNJ1	F:5' CAGAAACGTCGAAAGGTTCAAC3'<br>R:5' GTGCTGCCACAGGGACAAG3'	60	126
SYNM	F:5' TCCCTGGCACTGGTAGGGTA3'<br>R:5' AATGTTTGGGTGCTAAGGGATAA3'	60	177
SYT15	F:5' AGAGCTGTACAAGTTCCCGGA3'<br>R:5' ACTGCGGGTTGGAGGTTTT3'	60	242
SYCE1L	F:5' ACCCGCCAAGAAATAAAGG3'<br>R:5' CTCCACGGTGTAGCACAGACT3'	60	74
SYNDIG1L	F:5' CAGGCCAAGAAGGAAATACG3'<br>R:5' CTTGGTTCAGAGTTTACATACCGA3'	60	143
SDCBP	F:5' GAAATACGTGCAAATGTGGC3'<br>R:5' AACCAATGAGGCTGGAGAAT3'	60	258
SYCE1	F:5' AGCTGGTCAAGGCGACACTG3'<br>R:5' CCTGGCTGCGGAGAAAGAG3'	60	105
SYNDIG1	F:5' GCTGGCAAGAGGAATGGTTT3'<br>R:5' GATGATGTTGGGCCGGTAG3'	60	222
SYN2	F:5' CCTCAGTCGCCTCAATCTCG3'<br>R:5' TCGGTCATGTAGCCGTTGG3'	60	165
SYNGR4	F:5' CCGACGGCTACCAGAACAAG3'<br>R:5' CAGAGAACAGCCAGGATGAAGTC3'	60	211
SYNPO	F:5' AGCCGCAAATCCATGTTTACT3'<br>R:5' CCGCTTCTCATCCGCTGT3'	60	93
SYNRG	F:5' CGTTCTAGCCATGATAGCGG3'<br>R:5' TTGCAGGGTAGGTTGGTATGA3'	60	272
SYT3	F:5' TCTGGAGGGACATCGTGG3'<br>R:5' GTCCATCGCTTTGAGGTTAGA3'	60	134
STX2	F:5' GGAAGAAGCACAACCGAACG3'<br>R:5' GCCTGGATGACAAAGCGAGA3'	60	266
SYPL1	F:5' GCACATCTTGCTGTTGCCTTAT3'<br>R:5' AACTGTGATTCTATTTCCTGCTTTG3'	60	210

F: forward, R: reverse, Tm: melting temperature

Table S2. Genome-wide differential expression of lncRNAs in ASD

Differential expression	Bidirection	Exon sense-overlapping	Intergenic	Intron sense-overlapping	Natural antisense	Intronic antisense	Un-characterized	Subtotal
Up-regulated	113	210	1,053	69	492	392	78	2,407
Down-regulated	96	129	645	95	238	205	114	1,522
Total	209	339	1,698	164	730	597	192	3,929
