# Supplementary material for: Genome-wide differential expression of synaptic long noncoding RNAs in autism spectrum disorder
Source: Transl Psychiatry. 2015 Oct 20;5(10):e660–. doi: 10.1038/tp.2015.144 (PMC4930123; doi:10.1038/tp.2015.144)
Supplement: Supplementary Figure S1 [file tp2015144x2.pdf]

Figure S1

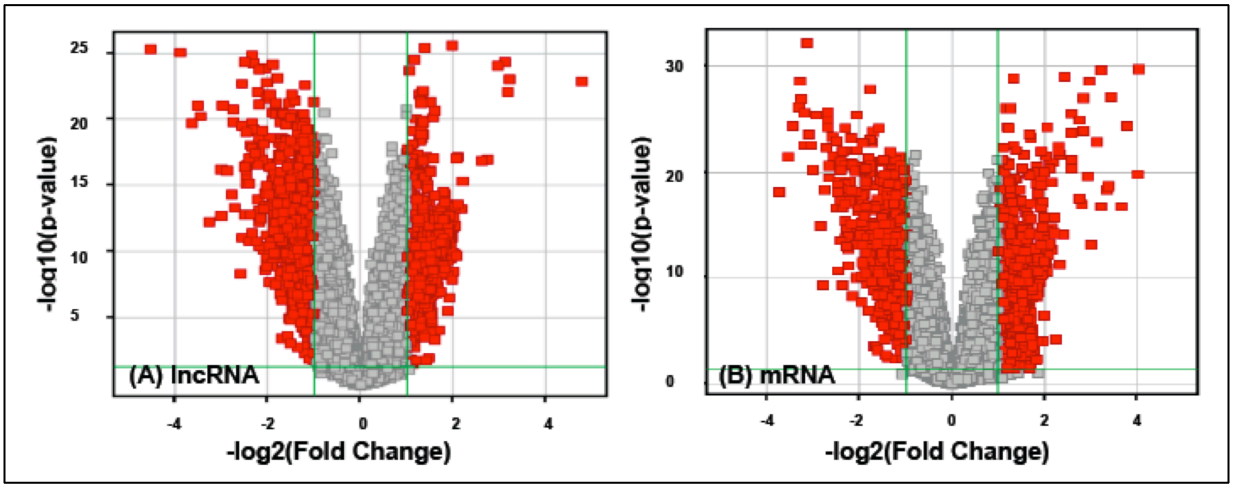

Figure S1: Volcano plots of differential expression of lncRNAs (A) and mRNAs (B): X-axis is fold change (log 2) and Y-axis is *p* value (-log 10). Up-regulated (X axis >0) or down-regulated (X axis <0) lncRNAs (red squares) were identified when fold change was set >2 folds [Log 2 (Fold change)] in ASD cases *vs*. control cases.
