# Supplementary material for: Genome-wide differential expression of synaptic long noncoding RNAs in autism spectrum disorder
Source: Transl Psychiatry. 2015 Oct 20;5(10):e660–. doi: 10.1038/tp.2015.144 (PMC4930123; doi:10.1038/tp.2015.144)
Supplement: Supplementary Figure S2 [file tp2015144x3.pdf]

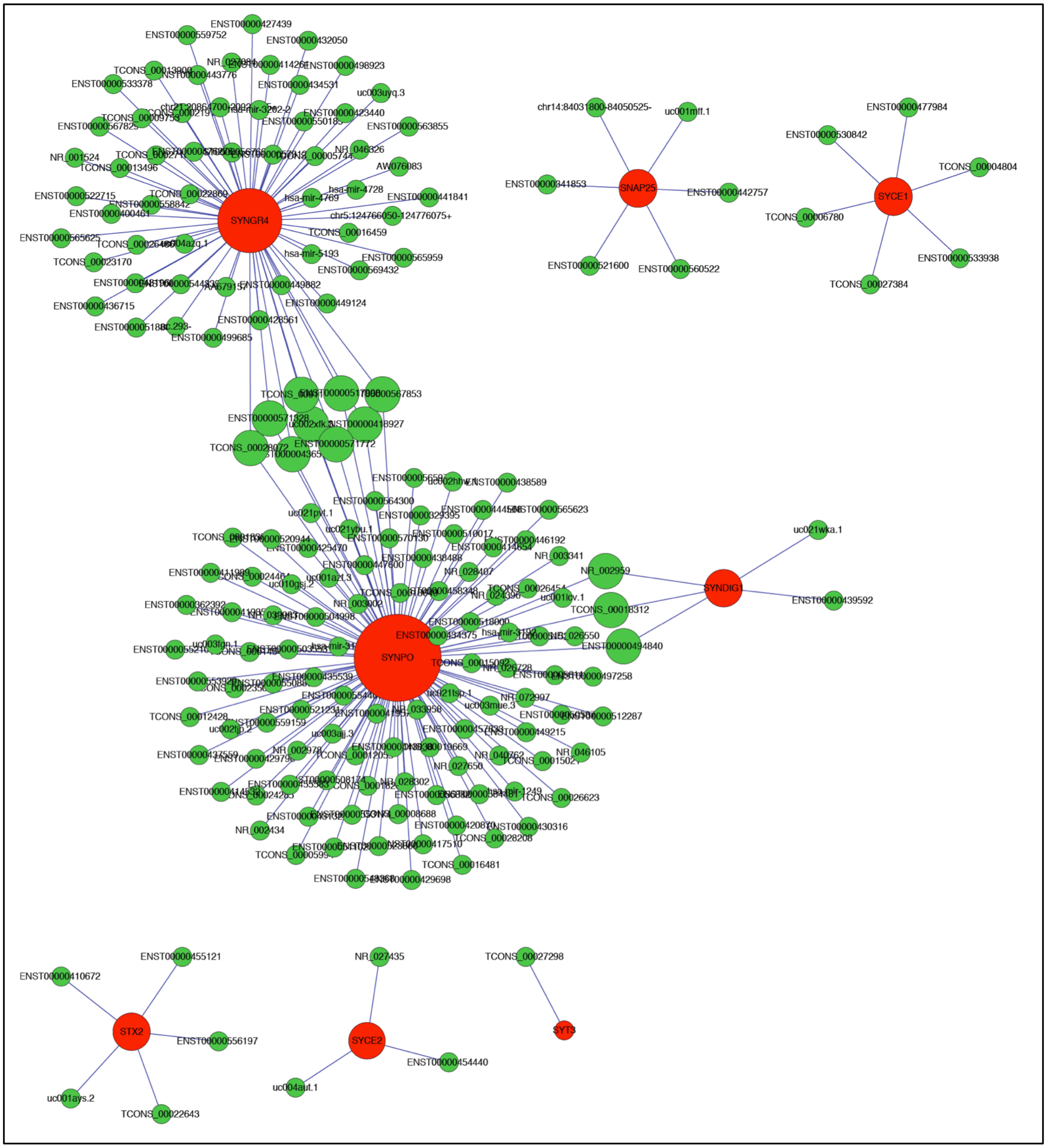

Figure S2. Network of synaptic mRNAs with lncRNAs identified from ASD: Synaptic mRNA (red ball) could be associated with either a single lncRNA (green ball), such as SYT3, or with numerous lncRNAs, such as SYNGR4 and SYNPO. Some lncRNA(s) may associate with two (or more) mRNAs. Knowing the relationship between mRNA and its associated lncRNA(s) may help in interpretation of possible regulatory outcome(s).
